# Quantitative approaches in climate change ecology

**DOI:** 10.1111/j.1365-2486.2011.02531.x

**Published:** 2011-12

**Authors:** Christopher J Brown, David S Schoeman, William J Sydeman, Keith Brander, Lauren B Buckley, Michael Burrows, Carlos M Duarte, Pippa J Moore, John M Pandolfi, Elvira Poloczanska, William Venables, Anthony J Richardson

**Affiliations:** * School of Biological Sciences, The University of QueenslandSt Lucia, QLD 4072, Australia; † Climate Adaptation Flagship, CSIRO Marine and Atmospheric ResearchEcosciences Precinct, Brisbane, QLD 4001, Australia; ‡ Environmental Science Research Institute, School of Environmental Sciences, University of UlsterColeraine, BT52 1SA, UK; § Department of Zoology, Nelson Mandela Metropolitan UniversityPO Box 77000, Port Elizabeth, 6031, South Africa; ¶ Farallon Institute for Advanced Ecosystem ResearchPO Box 750756, Petaluma, CA 94952, USA; ** National Institute of Aquatic Resources, Technical University of DenmarkCharlottenlund Castle, DK-2920, Charlottenlund, Denmark; †† Department of Biology, University of North CarolinaChapel Hill, NC 27566, USA; ‡‡ Scottish Association for Marine Science, Scottish Marine InstituteOban, Argyll, PA 37 1QA, UK; §§ Department of Global Change Research, IMEDEA (UIB-CSIC), Instituto Mediterráneo de Estudios Avanzados07190, Esporles, MallorcaSpain; ¶¶ The UWA Ocean Institute, University of Western Australia35 Stirling Highway, Crawley, WA 6009, Australia; *** Centre for Marine Ecosystems Research, Edith Cowan UniversityPerth, WA 6027, Australia; ††† Institute of Biological, Environmental and Rural Sciences, Aberystwyth UniversityAberystwyth, SY23 3DA, UK; ‡‡‡ Australian Research Council Centre of Excellence for Coral Reef Studies, School of Biological Sciences, The University of QueenslandSt. Lucia, QLD 4072, Australia; §§§ CSIRO Mathematics, Informatics and Statistics, Ecosciences PrecinctBrisbane, QLD 4001, Australia; ¶¶¶ Centre for Applications in Natural Resource Mathematics (CARM), School of Mathematics and Physics, University of QueenslandSt Lucia, QLD 4072, Australia

## Abstract

Contemporary impacts of anthropogenic climate change on ecosystems are increasingly being recognized. Documenting the extent of these impacts requires quantitative tools for analyses of ecological observations to distinguish climate impacts in noisy data and to understand interactions between climate variability and other drivers of change. To assist the development of reliable statistical approaches, we review the marine climate change literature and provide suggestions for quantitative approaches in climate change ecology. We compiled 267 peer-reviewed articles that examined relationships between climate change and marine ecological variables. Of the articles with time series data (*n* = 186), 75% used statistics to test for a dependency of ecological variables on climate variables. We identified several common weaknesses in statistical approaches, including marginalizing other important non-climate drivers of change, ignoring temporal and spatial autocorrelation, averaging across spatial patterns and not reporting key metrics. We provide a list of issues that need to be addressed to make inferences more defensible, including the consideration of (i) data limitations and the comparability of data sets; (ii) alternative mechanisms for change; (iii) appropriate response variables; (iv) a suitable model for the process under study; (v) temporal autocorrelation; (vi) spatial autocorrelation and patterns; and (vii) the reporting of rates of change. While the focus of our review was marine studies, these suggestions are equally applicable to terrestrial studies. Consideration of these suggestions will help advance global knowledge of climate impacts and understanding of the processes driving ecological change.

## Introduction

Although our knowledge of the impacts of anthropogenic climate change on biological systems is informed by the intersection of scientific theory, modelling, experiment and observation, it is only through observation that we can track the response of the biosphere to climate change. Understanding the extent of climate change impacts on ecosystems and their interactions with other anthropogenic stressors is a key requirement for informing policy debates on climate change and devising adaptive management responses (Harley *et al*., 2006; Edwards *et al*., 2010). Our knowledge of observed biological impacts of climate change is biased towards terrestrial systems (Richardson & Poloczanska, 2008); the analysis of observed climate impacts by the Intergovernmental Panel on Climate Change (2007) (their Figure 1.9) also indicates geographical imbalance in data availability.

Identifying the mechanisms driving change is especially challenging with marine biological data, because of short-term abiotic and biotic influences superimposed upon natural decadal climate cycles in the ocean-atmosphere system that can mask or accentuate climate change impacts (Hare & Mantua, 2000; Beaugrand *et al*., 2008; Möllmann *et al*., 2008). Anthropogenic drivers other than climate change, including eutrophication (Allen *et al*., 1998), fishing (Hsieh *et al*., 2008; Genner *et al*., 2010), pollution (Perry *et al*., 2005) and species introductions (Loebl *et al*., 2006) also interact with and complicate apparent ecological responses to climate change. Spatial variability in anthropogenic impacts and climate change (Halpern *et al*., 2008) mean that predictions from one region do not necessarily transfer to other regions. Furthermore, the availability of long time series suitable for generating baselines and for reliably testing hypotheses regarding climate impacts has been limited by funding and logistic issues (Duarte *et al*., 1992; Southward *et al*., 2005; Edwards *et al*., 2010). Despite these challenges, a long history of research has examined the influence of climate and other drivers on marine fisheries and ecosystem dynamics (ICES 1948, Colebrook, 1986; Ohman & Venrick, 2003; Southward *et al*., 2005). Climate change ecology has emerged from this research (e.g. Hawkins *et al*., 2003; Litzow & Ciannelli, 2007) and seeks to determine the extent of anthropogenic climate change impacts on ecosystems.

Appropriate statistical analyses are critical to ensure a sound basis for inferences made in climate change ecology. Many ecologists are trained in classical approaches more suited to testing effects in controlled experimental designs than in long-term observational data (Hobbs & Hilborn, 2006). Observational data are collected in space and time, so replicates may show strong dependences or autocorrelation effects and explanatory variables are often confounded (Legendre *et al*., 2002). Approaches that do not account for these issues may increase the risk of incorrect inferences and reduce power to detect relationships between climate variables and biological responses. Inference strength will also depend on the summary statistic chosen to represent biological responses, such as a species’ range edges or centre. Climate change ecology requires a greater awareness of statistical issues and the appropriate tools for obtaining reliable inferences from limited data sources.

Here, we provide suggestions for making defensible inferences in climate change ecology. We reviewed the literature on observed responses of biota to climate change to assess and describe current statistical practices in marine climate change ecology. On the basis of our assessment, we identify areas where the application of appropriate statistical approaches could be strengthened, including testing other potentially important drivers of change and their interactions with climate, consideration of temporal auto-correlation in time series, consideration of spatial heterogeneity and reporting of rates of change. We then provide suggestions for reliable statistical approaches that consider limitations of available data and highlight individual studies where statistical analyses were particularly innovative and reliable. We emphasize the strengths of individual studies to underscore lessons for the broader research community. While our focus is marine, our suggestions for statistical approaches are equally relevant for climate change research on land. Application of defensible statistical approaches will provide a more rigorous foundation for climate change ecology, improve predictive power and speed delivery of science to policy-makers and managers.

## Assessment of current statistical approaches in climate change ecology

We searched the peer-reviewed literature on climate change ecology for articles examining climate change impacts on the basis of observational studies. Our literature search was comprehensive and multi-faceted: extensive searches using Web of Science© and Google Scholar; citation searches; assessing every article published in key journals (Global Change Biology, Marine Ecology Progress Series, Progress in Oceanography, Global Ecology and Biogeography), analysis of reference lists in comprehensive reviews; assessment of studies from existing databases (Rosenzweig *et al*., 2008; Tasker, 2008; Wassmann *et al*., 2011) and our knowledge of various marine habitats. Studies were retained for analysis if the authors assessed the impacts of climate change on marine taxa, if there were data over multiple years after 1960 (when signals of anthropogenic climate change first became apparent), and if the primary climate variable investigated (e.g. temperature, sea ice) showed a change that the authors considered consistent with the physical impacts of anthropogenic climate change. We thus included studies with biological responses that were consistent or inconsistent with climate change. Only studies with observational data were considered for the review; therefore, studies with only experimental or modelling results were excluded. This process resulted in 267 studies published from 1991 to 2010, 186 of which used regularly sampled time series data. Time series generally started during or after the onset of anthropogenic warming in the 1960s (82% of time series studies in our review); however, several started before the 1960s (e.g. Ohman & Venrick, 2003; Reid *et al*., 2003, Southward *et al*., 2005). Data from palaeo-ecological studies dated as far back as 1700s (Field *et al*., 2006). For the time series studies, we recorded the type of statistical analysis used to relate climate and ecological variables, whether non-climatic factors were considered in analysis, and the methods used to deal with auto-correlation.

**Fig. 1:**
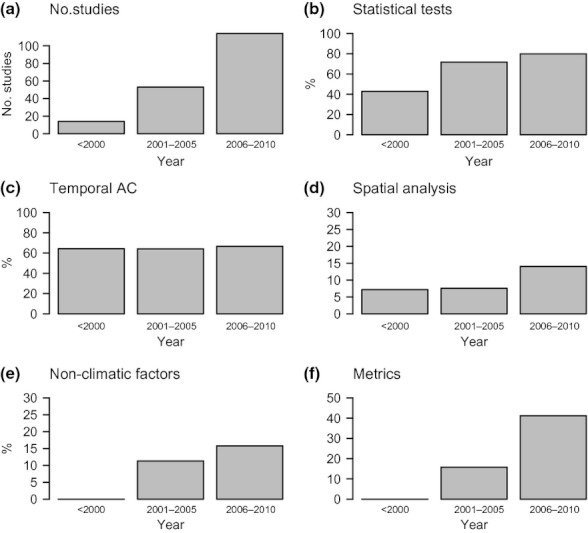
Attributes through time of marine studies in climate change ecology. (a) Number of studies. The remainder show percentage of studies (b) using statistical tests, (c) accounting for temporal autocorrelation, (d) using spatial analysis, (e) accommodating non-climatic factors and (f) reporting of metrics (distribution and phenology studies only).

The review showed an accelerating number of studies with time series data published in climate change ecology through time (Fig. 1a), consistent with the overall increase in climate change impacts studies published through time (Hoegh-Guldberg & Bruno, 2010). The proportion of studies using statistics to test relationships between climate and ecological variables has increased, doubling since before 2000 (Fig. 1b). The percentage of time series studies that accounts for or considers temporal auto-correlation remained around 65% (Fig. 1c). Both spatial analysis and modelling that accommodates non-climatic factors in addition to climate variables show increases over time, although rates of use remain low (Fig. 1d,e). Studies that report metrics on rates of change (e.g. km shifted per decade), useful for comparative studies and climate impacts syntheses, have also increased, although currently, only 41% of time series studies report these metrics (Fig. 1f). Together, the trends in use of statistics and reporting suggest that climate change ecologists have gradually been increasing their use of more reliable statistical methods, but overall, there is room to improve adoption and application of these methods.

To assess how statistical analyses might be currently perceived in the climate change ecology literature and whether those using more reliable statistics might be more highly cited, we recorded the number of citations each paper from the database received (on 12th February 2011) and tested whether citations were related to the statistical characteristics of the analysis. We used several binary predictors to reflect characteristics and included: whether temporal autocorrelation was accounted for; whether spatial analysis was conducted; whether metrics on rates of change were reported; whether multiple predictors were considered. Publication year was included as a covariate (using a cubic spline) to account for the growth of citations over time. We used a generalized linear model with negative binomial errors (Venables & Ripley, 2002) to model the effect of statistical characteristics on citation rate.

**Fig. 2:**
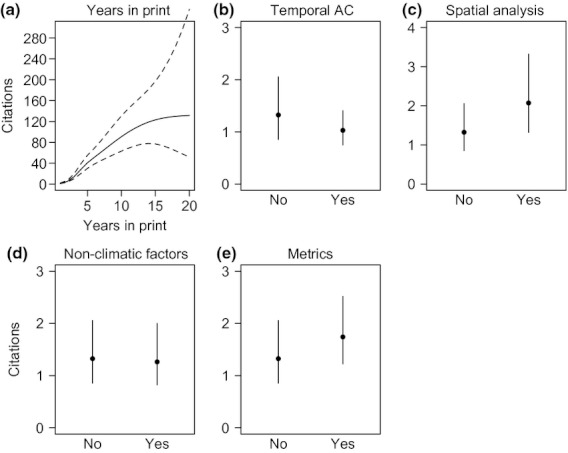
Effect size plots from a negative binomial GLM that analysed the number of citations for marine climate impact studies using time series data. Shown are effect sizes plots with 95% confidence intervals for (a) cubic spline for publication year (3 degrees of freedom) (b) whether temporal autocorrelation was considered; (c) whether spatial analysis was conducted; (d) whether alternative non-climatic factors were considered in analysis; and (e) whether metrics were reported. For each plot parameters for other categorical variables were fixed to ‘No’ and 1 year in print.

Generally, it might be expected that more reliable statistical approaches and reporting of metrics would improve a study's usefulness in the literature and hence the citation rate. Indeed, studies that use spatial methods may be cited slightly more often (Fig. 2c). Furthermore, studies that reported metrics on rates of change may also have slightly higher citation rates, suggesting that these studies are used more often in the literature because of the ease of comparison (Fig. 2e). Relative to the effect of years in print, the improvement in citations was slight and studies that accounted for temporal autocorrelation or modelled multiple factors were not cited more often (Fig. 2b,d).

The results of our review and citation analysis may indicate both inadequate awareness of appropriate statistical techniques for analysis of observational data and a lack of suitable data to support more sophisticated analyses. That studies employing more reliable statistical approaches were not more highly cited indicates a need for greater scrutiny of statistical approaches in marine climate change ecology. Data limitations are also important, and greater funding of marine ecological time series would allow a more comprehensive analysis of climate change impacts (Duarte *et al*., 1992; Southward *et al*., 2005; Edwards *et al*., 2010). Nevertheless, there are studies in the marine climate change ecology literature and from other research areas that illustrate a range of effective statistical approaches for maximizing the utility of available data. In the following sections, we use these studies as examples of how to make the most of available data, address statistical issues and as a basis for suggesting reliable methods for statistical analysis in climate change ecology.

## Data requirements for assessing climate change impacts

Strongest inferences on impacts of climate change require observational data that cover long time spans and large spatial scales (Parmesan *et al*., 2011). However, funding constraints on the extent of data collection have limited the length of time series and their spatial extent (e.g. Southward *et al*., 2005; Edwards *et al*., 2010). Some examples of long time series that have persisted through funding cycles are the Continuous Plankton Recorder survey in the North Atlantic and North Pacific (Colebrook, 1986; Reid *et al*., 2003); the California Cooperative Oceanic Fisheries Investigations in the Californian Current (Ohman & Venrick, 2003); and fish, zooplankton and rocky shore surveys conducted from Plymouth, UK (Southward *et al*., 1995, 2005).

Longer and higher frequency time series data provide greater opportunities to investigate the effects of climate and anthropogenic impacts on ecosystems. In the English Channel, long-term cycles of rocky shore and pelagic fish communities coincide with cycles of cold and warm periods, providing strong evidence that modern shifts to warmer-water communities are a consequence of warming in the region (Hawkins *et al*., 2003; Southward *et al*., 2005). Likewise, longer time series are required to provide baselines for assessing the impacts of anthropogenic climate change. Data from the English Channel demonstrate that while communities have cycled naturally over long periods, recent changes have exceeded those observed in the last warm period, in the 1950s, and are probably a result of anthropogenic climate change (Mieszkowska *et al*., 2007). Distinguishing the effects of multiple drivers also requires data that allow contrasts between strengths of each driver, because if the drivers co-vary strongly, it will be difficult to determine their individual effects. In this case, longer time series or data collected over a larger spatial scale potentially provide greater opportunities for sampling contrasts.

### Comparing historical and contemporary data sets

**Fig. 3:**
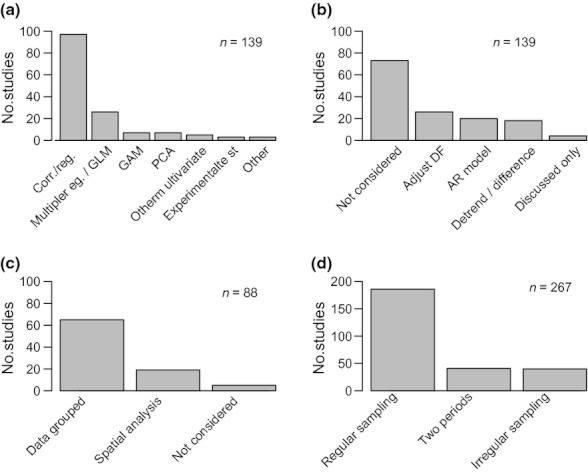
Number of studies that (a) use different methods for relating climate and biological time series; (b) use different methods for adjusting for temporal auto-correlation; (c) group data, use spatial analysis or do not consider spatial autocorrelation, and (d) use different data types. The number of relevant studies included for each figure is indicated. Studies that applied multiple different types of methods were counted once for each method. GLM, generalized linear model; GAM, generalized additive model; PCA, principal components analysis.

Baselines for assessing climate impacts for data-poor regions or taxa can be obtained by conducting surveys in sites where historical data are available and comparisons can be made between present and historical data. While most studies in our database were based on regularly collected samples, samples collected at irregular intervals or those comparing two distinct periods in time were also common (Fig. 3d).

Data collection designs that pre-date the advent of modern statistical approaches pose challenges to comparisons with contemporary data sets (Tingley & Beissinger, 2009). Differences in survey methods between past and present programs may confound biological responses to climate change. Similarly, a major problem for range-shift studies is determining the difference between true absences of species at a site and false absences that result from missed detection or historical records restricted to few species (Tingley & Beissinger, 2009).

Nevertheless, historical data are valuable and should not be discarded because they pose challenges to analysis. Indeed, appropriate statistical approaches can assist with the integration of old and contemporary data. Often, careful consideration of changes in data collection methodology can identify biases that can then be factored out in analysis, for instance, by comparing changes in relative rather than absolute abundances of species (Fodrie *et al*., 2010). Tingley & Beissinger (2009) review approaches for comparisons of historical and contemporary data in range shift studies. In particular, methods for estimating detection probability of a species are useful for distinguishing false and true absences to provide more accurate mapping of range shifts. The lack of temporal continuity in comparisons with historical data also limits the ability to analyse the relationship between climate variables and species distribution. Using historical and contemporary data on seaweed distribution, Lima *et al*. (2007) apply a randomization procedure to explore whether range differences between the two time-periods are significantly greater than would be expected on the basis of distances between modern sub-populations. This approach allows Lima *et al*. (2007) to make stronger inferences about observed changes in range size.

Caution is required in the interpretation of differences between two points in time because patterns of variability in the intervening years are not captured. For instance, in the North-East Atlantic, comparisons between the 1960s and 2005 exaggerate warming because of unusually cold years in the 1960s (Hawkins *et al*., 2003; Southward *et al*., 2005). Although two-point comparisons have been applied to a broad range of taxa in the literature, the most reliable comparisons will come from taxa with low inter-annual variability relative to the magnitude of change between the two time periods. The relative magnitude of inter-annual variability can sometimes be estimated by comparison to species with similar ecology or directly from data if multiple years are available at analysis start or end points (e.g. Sagarin *et al*., 1999 had multiple years of data, from 1931–1933 and 1993–1996). A further disadvantage of point comparisons is the low power for discriminating among multiple drivers of change because most drivers will have changed between historical and present studies.

Nevertheless, point comparison analysis can at least partially overcome the disadvantages of low temporal resolution by including data on many species. For example, Fodrie *et al*. (2010) repeated historical surveys and compared abundances of fish in seagrass meadows between the 1970s and the present day. The community analysis revealed that cold-water species were less likely and warm-water species more likely to be observed in the present day, a result consistent with mechanisms of a climate change impact. Furthermore, a *t*-test comparing the pooled abundance of warm-water species between the historical period and the present day confirmed that warm-water species had increased in relative abundance. A final *t*-test showed a significant warming in regional temperature. It is important to note that the historical and recent period studied by Fodrie *et al*. (2010) were sufficiently separated in time (1970s vs. 2000s) to allow for a clear warming signal.

### Retrospective data in climate impact studies

Given the relative paucity of long biological and ecological time series, retrospective methods for obtaining data to test for impacts of climate change provide a rich and relatively untapped resource. In particular, fast sedimentation rates in many areas of the ocean preserve micro-organisms over centuries to millennia and these sedimentary records can be examined in relation to recent climate changes. We found 13 retrospective studies in the literature review of climate change ecology and these included studies of fish otoliths (Thresher *et al*., 2007), calcifying plankton from sediment cores (Field *et al*., 2006) and coral cores (De'ath *et al*., 2009). Retrospective studies have great potential importance for assessing shifts in patterns of biological variability before and after the onset of warming, because they date to before detection of global warming signals in the 1960s.

Field *et al*. (2006) used sediment cores from the Californian Current region to examine long-term changes in the planktonic foraminifera community. Foraminfera preserve well in sedimentary records because of their calcium carbonate shell. The time series dated back to before global industrialization and demonstrated a shift from a cold-water community to a warm-water community around the 1970s that was unprecedented in the past 200 years. Furthermore, the shift in community structure showed a strong correlation with reconstructions of sea surface temperature.

A major shortcoming of many retrospective studies is the limited number of samples or sediment cores that can be obtained. So while temporal coverage may be high, spatial or sample-based replication may be low. The Field *et al*. (2006) study was based on just a few sediment cores, due to the difficulty of obtaining deep-sea cores. This limits the ability to examine temporal patterns in climate impacts over broad spatial scales using retrospective analyses.

## Addressing statistical issues

A major challenge in statistical analysis is simultaneously minimizing risks of attributing causality to simple associative relationships and of missing relationships that are the result of real ecological processes. Properly formulated statistical tests of the relationship between the ecological variable of interest and a variable indicative of climate change help minimize these risks. Of the time series studies we reviewed, 47 (25%) did not use statistical tests to relate ecological trends to climate variables.

Aside from using properly formulated statistical tests, these errors can be minimized by formulating plausible mechanisms for the form, direction and magnitude of biological change. An understanding of mechanisms helps to build confidence that statistically weak but mechanistically plausible relationships are sound (for instance, when data are limited) and, similarly, helps to exclude statistically significant but spurious relationships. For example, inferential strength from observational studies can be improved by coupling the study with appropriate experimental studies (three studies in our review, Chevaldonné & Lejeusne, 2003; Iglesias-Rodriguez *et al*., 2008; Halloran *et al*., 2008). Chevaldonné & Lejeusne (2003) showed long-term declines in cold-water mysid abundances in Mediterranean caves attributable to warming. They were able to grow these mysids in the laboratory to demonstrate that contemporary warming was beyond their preferred temperature range. This approach is potentially a powerful way to investigate the mechanisms driving climate responses in organisms amenable to experimentation (e.g. intertidal invertebrates, macro-algae and corals). Hewitt *et al*. (2007) provide a comprehensive review on strategies for integrating small-scale, manipulative studies with large-scale correlative studies.

### Accommodating multiple factors in analyses

When investigating ecosystem change, a host of anthropogenic impacts (including climate) and natural dynamics are confounded, complicating interpretation and potentially leading to spurious conclusions when important drivers are not included in analysis. Statistical analyses in our review were predominately univariate (correlation or simple linear regression, Fig. 3a), which do not allow consideration of multiple factors and their interactions. Only 24 time series studies (13%) in the literature reviewed explicitly considered factors other than climatic variables in statistical analysis (e.g. Hsieh *et al*., 2008; Poloczanska *et al*., 2008). At the simplest and coarsest level, the often-strong trends in the primary climate variables considered (temperature, sea ice) can be correlated with increases in anthropogenic threats of eutrophication, fishing and pollution, as increases in both CO_2_ emissions and human threats are a consequence of increases in human population and activity (Halpern *et al*., 2008). The lack of inclusion of alternative factors also implies that key interactions between drivers, which could be important for predicting and managing ecosystem responses to climate change, are not being addressed.

Of the studies that consider multiple factors in analysis, generalized linear modelling (including multiple regression), a method common in the broader ecological literature, was the most popular (Fig. 3a, e.g. Dulvy *et al*., 2008). Generalized additive models were also used by seven studies. There is already an extensive literature discussing application of these methods to modelling multiple factors and their interactions (see Table 1 for more details) and therefore we describe two examples below where innovative approaches were used to understand the influence of multiple explanatory variables.

**Table 1:** Summary of statistical approaches described in the text, with references, and appropriate routines in the free statistical package R. See http://cran.r-project.org/ to obtain R, its packages and user guides (R Development Core Team, 2010)

Statistical consideration	Reasons to consider	Statistical solutions	Examples: climate change ecology	References for methodology	R guide
Multiple factors influence response	Ignoring multiple factors may result in attributing biological change to wrong driver or missing interactions	Multiple regression, generalized multiple regression	Dulvy *et al*. (2008)	Crawley (2005)	Functions lm() and glm()
		Generalized additive modelling	De'ath *et al*. (2009), Mueter & Litzow (2008)	Wood (2006), Zuur *et al*. (2009)	‘mgcv’ package
		Path analysis/structural equation modelling	Poloczanska *et al*. (2008), Kirby & Beaugrand (2009)	Grace (2006)	‘sem’ package
		Community analysis: compare species with different traits	Hsieh *et al*. (2008), Fodrie *et al*. (2010), Field *et al*. (2006)	NA	NA
Temporal autocorrelation and spurious trends	Ignoring autocorrelation may result in false attribution	Detecting autocorrelation patterns		Zuur *et al*. (2009)	Function ‘acf()’
		Differencing and detrending	Juanes *et al*. (2004), Votier *et al*. (2009)	Pyper & Peterman (1998)	Function ‘diff(y)’ for differencing, use ‘resid(lm(y∼x))’ for detrending y on x.
		Modify degrees of freedom	Richardson & Schoeman (2004), Möllmann *et al*. (2008), Beaugrand & Kirby (2010), Kirby & Beaugrand (2009), Hermant *et al*. (2010), Wiafe *et al*. (2008)	Pyper & Peterman (1998)	None known
Temporal autocorrelation and spurious trends		Autoregressive models	Brodeur *et al*. (2008)	Zuur *et al*. (2009)	Function ‘gls()’ in the ‘nlme’ package and function ‘ar()’
		Autoregressive moving-average models and Autoregressive integrated moving-average models	None	Legendre & Legendre (1998), Zuur *et al*. (2009)	Function ‘gls()’ in the ‘nlme’ package and function ‘arima()’
		Cointegration	None	Kirchgässner & Wolters (2007)	Package ‘urca’
Spatial patterns and autocorrelation	Ignoring autocorrelation may result in false attribution, aggregating over spatial patterns losses information	Identifying spatial autocorrelation in continuous data	Mueter & Litzow (2008)	Legendre & Legendre (1998)	Package ‘spdep’
		Model spatial patterns using meta-analysis	Richardson & Schoeman (2004)	Worm & Myers (2003)	None known
		Model spatial patterns using linear modelling or generalized additive modelling	Mueter & Litzow (2008), De'ath *et al*. (2009)	Wood (2006), Diggle & Ribeiro (2007)	‘stats’ package and ‘mgcv’ package
		Model discrete sites using random effects	De'ath *et al*. (2009), Forcada *et al*. (2006)	Zuur *et al*. (2009), Pinheiro & Bates (2000)	Packages ‘nlme’ and ‘lme4’ for linear and generalized linear modelling and ‘mgcv’ for generalized additive modelling
		Model spatial autocorrelation	Mueter & Litzow (2008)	Zuur *et al*. (2009), Pinheiro & Bates (2000)	Package ‘nlme’
Temporal cycles and variance	Climate impacts may manifest as changes in temporal variance or cycles	Models of temporal variance	Beaugrand *et al*. (2008)	NA	NA
		Wavelets	Jenouvrier *et al*. (2005)	Torrence & Compo (1998)	Package ‘Rwave’
		Fourier transform	NA	Legendre & Legendre (1998)	Function ‘fft()’

NA, not available.

Along with climate change, fishing pressure is arguably the most widespread human impact on marine ecosystems (Halpern *et al*., 2008). Unfortunately, data on exploitation rates often do not exist or are difficult to obtain (but see Dulvy *et al*., 2008; Genner *et al*., 2010). Hsieh *et al*. (2008) used a novel approach to overcome the lack of data on temporal dynamics of exploitation rate. They analysed changes in the distribution of larval fish under ocean warming. To account for exploitation rates, they conducted a comparative analysis of the effects of climate on the spatial distribution of exploited and unexploited fish species. By comparing impacts of climate on species with similar life-history traits, they were able to partly control for effects introduced by differences among species, and focus on impacts of exploitation and climate on fish distribution. Importantly, their analysis demonstrated a synergism between climate and fishing impacts, with exploited species being more sensitive to climate-driven range shifts than unexploited species. As more studies incorporate climate change and other human threats into their statistical models, we should develop a greater understanding of how we can manage our marine systems to minimize the effect of climate change.

Considering multiple factors may also help test competing hypotheses regarding the structure of underlying relationships between a species, climate and its ecosystem. Analysis of multiple hypotheses is also important for assessing uncertainty in the outcomes of climate change impacts. Hobbs & Hilborn (2006) provide a useful guide on how multiple model formulations can be tested against observed data. One approach for multi-model inference is to develop structured models using path analysis and then to compare their ability to predict observations (Table 1). For example, Poloczanska *et al*. (2008) investigated the recruitment of two barnacle species in relation to warming temperatures by constructing a hierarchy of models of increasing complexity. Different models considered the response of each species to warming individually and including interactions between species such as resource and interference competition. They found that climate change may be impacting directly one species, which was, in turn, impacting its competitor via interference competition. In this case, testing the ability of different models to predict observations provided a more reliable assessment of the climate change signal by identifying both the direct and indirect mechanisms of the climate change impact.

### Identifying spurious relationships and accounting for auto-correlation in biological data

Temporal and spatial autocorrelation arise from non-independence of observations and are a common feature of time series and geographical studies (Legendre *et al*., 2002). Autocorrelation can be caused by factors exogenous to the variables of interest, such as unknown environmental effects on population size, and factors endogenous to the variables of interest, such as the effect of intra-specific competition species on population size. Temporal autocorrelation is commonly strong in marine ecological data. For instance, the same individuals will be counted in multiple years in population counts of longer lived species and data from heavily fished species are often strongly autocorrelated due to effects of economic development of fishing fleets and management regimes. Autocorrelation can occur over multiple time-scales in a dataset, including seasonal patterns at short time-scales and long-term trends due to gradual changes in observation methods or evolutionary change in the species studied. Similarly, spatial autocorrelation can occur at a range of scales. For instance, small-scale spatial autocorrelation may be observed in species that aggregate to breed or where individuals of a species disperse to avoid competition, and large-scale autocorrelation may be present if important environmental gradients are unspecified in models.

A basic assumption of most inferential statistical tests – that residuals are independently and identically distributed – will be violated if residuals are autocorrelated. Thus, autocorrelation that is unaccounted for can result in misleading inferences. In autocorrelated data, each measurement does not contribute a full degree of freedom to the analysis, so degrees of freedom in statistical tests are over-estimated, and this inflates the Type-I error rate (falsely rejecting true null hypotheses). For instance, Worm & Myers (2003) estimated effective degrees of freedom from fisheries data, and found that degrees of freedom may be inflated by up to six times in cod–shrimp correlations if autocorrelation is not considered. In many cases, exogenous autocorrelation may be removed if appropriate covariates are included in the model. Alternatively, it is necessary either to explicitly model the autocorrelation structure, or to adjust degrees of freedom in statistical tests (i.e. estimate the effective sample size, given the autocorrelation) on the basis of the autocorrelation structure (see Table 1 for how methods on detecting autocorrelation).

In the review of the climate change ecology literature, 68 studies (49%) analysing biological changes over time considered temporal autocorrelation (Fig. 3b). Further, 19 studies (21%) with data at multiple locations made explicit use of spatial methods that either accounted for spatial autocorrelation or modelled covariates spatially (Fig. 3c). Most studies grouped spatial data, thus not only avoiding issues with spatial autocorrelation, but also potentially removing important ecological patterns from analysis. In the following section, we discuss examples from climate change ecology that deal with temporal autocorrelation, spatial autocorrelation and spatial patterns in statistical analyses (for details of methods see Table 1). Many methods are common to both types of autocorrelation and therefore we provide references for further details.

### Accounting for temporal autocorrelation and spurious relationships

The simplest approach to deal with temporal autocorrelation is to remove autocorrelation by differencing the climate and biological data series (subtract each data point from next data point in the time series, Table 1) over the autocorrelation time-scales prior to statistical analysis (Pyper & Peterman, 1998). De-trending (subtracting the long-term trend from each data point, Table 1) may also be desirable to remove shared long-term trends because time series commonly trend without a causal link. However, removing trends can reduce the power to detect real relationships (Pyper & Peterman, 1998) and, in some cases, differencing or detrending can increase the autocorrelation in a dataset. For instance, if measurements in a time series are independent, detrending the time series will create a dependency among data points. Historically, such data transformations were used to obtain datasets that met the assumptions of the statistical tests available. The advent of modern model-based approaches that accommodate autocorrelation processes provides the opportunity to avoid the shortcomings of data transformations.

When the climate–biological relationship is expected to operate over longer time-scales, the data can be smoothed using a filter before conducting statistical tests such as regression. Smoothing reduces the influence of short-term variability that is not of primary interest. For instance, Litzow & Ciannelli (2007) use a smoother to examine the inter-annual relationships among abundances of predators, prey and physical conditions, and Dulvy *et al*. (2008) use a smoothing filter on their environmental data to capture the integrated influence of the environment on species’ distribution over several years.

A method of accounting for autocorrelation in correlation tests that has gained particular favour in studies of climate impacts on plankton and fish communities (24 studies in all, e.g. Richardson & Schoeman, 2004; Litzow & Ciannelli, 2007; Möllmann *et al*., 2008; Nye *et al*., 2009; Beaugrand & Kirby, 2010), is to explicitly adjust the degrees of freedom downwards relative to the amount of temporal autocorrelation in the time series, before calculating significance levels (Table 1). Pyper & Peterman (1998) used simulated data to test error rates for different methods of adjusting the degrees of freedom on a significance test of correlation coefficients. Their simulations indicated that methods for adjusting degrees of freedom reduce the risk of falsely attributing significance to a relationship without the loss of power that de-trending the data may cause. Thus, despite the greater technical knowledge required, these approaches are generally preferable to de-trending the data before testing a correlation. Dale & Fortin (2009) describe two straightforward methods for undertaking such analyses in a spatial context.

Potentially, the most powerful procedure for accounting for auto-correlation is to use an auto-regressive model (Table 1). An auto-regression can be advantageous over correlation approaches with adjusted degrees of freedom because regression allows estimation of the rate of change of the biological variable and for multiple covariates to be considered simultaneously. Estimates of the autocorrelation structure may also suggest mechanisms for its cause. Researchers should carefully consider the mechanisms behind the proposed term, rather than choosing an auto-regressive model based on goodness of fit alone, because adding an auto-regressive term to a model can reduce the power to detect a change. For example, Brodeur *et al*. (2008) consider the effect of sea ice extent and temperature on the biomass of jellyfish in the Bering Sea. Autocorrelation in jellyfish biomass from 1 year to the next was expected because the biomass of jellyfish in 1 year should depend upon the biomass of animals reproducing in the previous year. Brodeur *et al*. (2008) used generalized additive modelling to build multiple models that regress jellyfish biomass against climate variables, whilst accounting for autocorrelation by using 1-year lagged jellyfish biomass as a factor in the model. They then compared the ability of the models to predict data using a generalized cross-validation approach (Wood, 2006). As expected, jellyfish biomass in 1 year was strongly positively associated with biomass in the preceding year. Sea ice and temperature were also correlated with jellyfish biomass after accounting for the autocorrelation effect. Their analysis thus revealed potential interactions between climate and jellyfish growth, without concerns that significance would be spuriously inflated by temporal autocorrelation.

A major source of new methods for time series analysis has been economics. The concept of cointegration was developed by econometricians to allow inferences on causality of long-term relationships without the loss of power associated with differencing time series to obtain stationarity (Engle & Granger, 1987). Two time series are said to be cointegrated in the first order if the residuals from a linear combination of the time series are stationary (the mean does not change through time). Tests for cointegration distinguish between time series with independent stochastic trends and those that share a long-term relationship (Table 1). For instance, consider two time series for temperature and fish recruitment. If both time series have an increasing trend, we might difference the time series and correlate the resulting series to test for a relationship. However, if temperature really does drive long-term trends in recruitment, then differencing the time series will reduce the power to detect a real causal effect. Alternatively, we could test for cointegration of the time series. Cointegration of the time series would imply a causal driver of the shared long-term trend between the time series, whereas if the time series are not cointegrated, then we have not properly accounted for a causal relationship.

Cointegration has also been extended to multivariate and higher order analysis of time series with multiple orders of integration (Kirchgässner & Wolters, 2007). Cointegration proved extremely useful in the analysis of economic time series, with Engle and Granger awarded the 2003 Nobel Memorial Prize in Economic Science for their contribution to time series analysis. It is thus surprising that this approach has been almost entirely ignored in ecological time series analysis. Interested readers should refer to Kirchgässner & Wolters (2007) for an introduction to cointegration methods accessible to ecologists.

### Accommodating spatial patterns and autocorrelation in biological data

One approach to account for spatial patterns in data is to perform a meta-analysis of study regions (Worm & Myers, 2003). Richardson & Schoeman (2004) analysed the correlation between phytoplankton abundance and sea surface temperature in a 45-year time series for multiple areas of the North-East Atlantic. They found no relationship in most areas when significance tests were adjusted for temporal autocorrelation. However, the study covered a gradient of mean annual temperature ranging from about 6 to 20 °C. Thus, Richardson & Schoeman (2004) used meta-analysis to inspect the correlation between mean annual temperature in each region and the temporal abundance–temperature correlation. The meta-analysis showed a significant negative correlation, implying that temperature rise positively impacted phytoplankton abundance in cold regions, negatively impacted abundance in warm regions and had little effect in intermediate regions. Such a result was consistent with the proposed mechanism for climate impacts, with phytoplankton growth being limited by low temperature in cold regions and thermal stratification in warm regions. Thus, the analysis of spatial patterns in this study revealed ecologically important signals, which would have remained hidden if the data were aggregated.

An alternative approach to modelling spatial patterns is to include spatial covariates in multiple regression or generalized additive models (Table 1). De'ath *et al*. (2009) analysed data on coral growth and calcification using coral cores from 69 reefs across the Great Barrier Reef, Australia. By measuring growth rings in coral cores, calcification rates as far back as 1572 could be estimated. De'ath *et al*. (2009) used a generalized additive model to determine whether there were long-term trends in calcification and if calcification changes could be related to temperature changes. Their study area covered a significant spatial temperature gradient, so they divided temperature into spatial and temporal covariates. Thus, they were able to distinguish between the spatial effect of higher calcification rates in warmer regions and the temporal effect of more variable calcification rates during warmer years.

Spatial patterns in data cannot always be removed by including additional covariates in analysis. Where this spatial autocorrelation occurs, it should be considered in statistical tests (Table 1). Richardson & Schoeman (2004) took a simple approach and reduced spatial autocorrelation in their meta-analysis by using only spatially discontinuous sites. The downsides of this approach are that data are excluded from analysis and that it cannot account for spatial autocorrelation occurring across larger areas. As with temporal autocorrelation, spatial autocorrelation can also be estimated and accounted for in tests. Mueter & Litzow (2008) compared changes in the distribution of abundance of fish species between two time periods. They fitted models of spatial autocorrelation to their data (Pinheiro & Bates, 2000) and found a weak spatial autocorrelation that might inflate standard errors in statistical tests by 10%. Thus, to reduce the risk of detecting spuriously significant distribution change, they added an additional 10% to the standard errors before testing.

Ideally, spatial autocorrelation would be included explicitly as a process in a spatio-temporal model. Examples include accounting for spatial structure in error terms or response variables by adjusting the variance–covariance matrices in regressions or conducting geographically weighted regressions (Kissling & Carl, 2008). We found no examples in the literature we reviewed probably because such models are technically challenging to develop. Data requirements can also be intensive, with a need for data across numerous locations. For the technically inclined, Diggle & Ribeiro (2007) provide a starting point for geostatistical analysis.

Often, biological data are collected at discrete locations, where samples from the same location are expected to be more similar than samples from different locations, although the likely causes of sample dependencies are unknown. De'ath *et al*.'s (2009) data were replicated at discrete locations, with multiple calcification measurements from each core and multiple cores at each reef. If replicates from the same location are treated as independent samples, they might spuriously inflate the degrees of freedom in statistical tests. Alternatively, pooling samples would considerably reduce the sample size and the power to detect causal relationships (Venables & Ripley, 2002; Table 1). De'ath *et al*. (2009) accounted for the nested structure in the data by including random effects for cores and reefs in their generalized additive model. Calcification measurements from the same core were treated as random deviates from an overall core mean value and similarly for reefs. Accounting for the nested structure allowed reliable inferences on the temporal and spatial effects of temperature while preserving the power of the analysis. Random effects analyses are also useful when data are too limited and spatially unresolved to properly estimate spatial autocorrelation in a geo-statistical analysis.

### Modelling changes in variability, cycles and periods

Most cases discussed so far have focussed on the effect of climate change on trends in ecological response variables. Climate impacts may also be detected through the examination of changes in the variability of ecological responses, including changes in the magnitude, frequency and period of ecological responses. Beaugrand *et al*. (2008) examined variability in metrics for cod recruitment and plankton community structure, size and diversity in the North Atlantic. Spatial analysis of these metrics revealed increased variability coinciding near the mean annual 10 °C isotherm, potentially indicating an ecological threshold separating different community types. Examination of the temporal variance in the community metrics demonstrated increased community variance in an area as the water warmed and the 10 °C isotherm moved polewards through an area. This increase in variance may indicate a shift in community composition to one that represents a more southerly biogeographical province.

Large-scale climate cycles may also drive periodic biological patterns. Sophisticated approaches have been developed by physical scientists that allow time series to be decomposed into their component cycles. These methods may be particularly useful for the analysis of highly temporally resolved long-term marine ecological data, and allow the separation of long-term trends from decadal cycles in the ocean. One flexible approach is wavelet analysis (Torrence & Compo, 1998), which decomposes a time series into time and frequency domains, thus allowing examination of the dynamics of dominant cycles in the data (Table 1). Jenouvrier *et al*. (2005) applied wavelet analysis to time series of seabird abundance, breeding success and environmental variables thought to affect seabird foraging success. They showed that in the early 1980s, there was a shift in the periodicity of both the seabird time series and the environmental time series, coincident with large-scale ocean warming. Thus, Jenouvrier *et al*. (2005) were able to detect changes in population variability potentially driven by climate warming that might not have been detected by examining trends in abundance or breeding success.

### Metrics of phenology and distribution

The interpretation of climate impacts may often be assisted by deriving metrics of biological responses from raw observations that are readily associated with climate change. Overall, climate change is expected to lead to a polewards migration of species’ biogeographical ranges and an advance in the timing of phenological events (e.g. reproduction, migration). Derived metrics have proved useful for meta-analyses in climate change ecology. In particular, reports of rates of change in distribution (e.g. km decade^−1^ or km °C^−1^) or phenology (days decade^−1^, days °C^−1^) are easily incorporated into global meta-analyses and syntheses, including those by the IPCC (2007). Despite the benefits, reporting of these metrics is still not widespread in marine climate change ecology (18 out of 55 phenology and distribution studies with regularly sampled data reported metrics of change).

There is a range of analogous response metrics for phenology or distribution, which have similar statistical strengths and weaknesses. In studies of phenology, metrics include timing of an event on the basis of a single individual (e.g. arrival of the first individual), the mean or median timing of the event, the timing of the last event (e.g. departure of the last individual), or the duration of the event. Similarly, analyses of distribution shifts may use the range edges, range centre or range size as an indicator of range shift. The statistic used to represent the range or date changes should be carefully considered.

There are a suite of indicators that are reliant upon single individuals or single sites, such as the first individual to breed, or the northernmost sighted individual. These are statistically weak indicators of phenological change and distribution shifts because they are dependent upon only a single individual or site and ignore the majority of the population. More reliable metrics of changes in phenology and distribution are based on data on populations, such as recoding of the distribution of individual breeding dates in a population, abundance across the range or presence at different sites. In these cases, quantiles can be used to indicate the beginning of an event or the edge of a range. For instance, Juanes *et al*. (2004) analysed the dates of arrival for salmon to breeding streams using the cumulative dates of arrival of 25%, 50% and 75% of all fish, and Greve *et al*. (2005) analysed the start and end of the season using 15% and 85% of the annual cumulative abundance thresholds for plankton.

Commonly, the spread of abundance across a species’ distribution has been assumed to be normal, on the basis of early macro-ecological theory (Brown, 1984). In this circumstance, mean spatial location (e.g. mean latitude of occurrence) would be an appropriate metric for the distribution centre. In reality, distributions of abundance may often be non-normal, in which case, the most appropriate metric for representing a distribution centre will depend upon the spatial arrangement of site presences and abundance (Sagarin *et al*., 2006). For instance, Hsieh *et al*. (2009) analysed changes in the mean and median distributions of larval fish, and found that changes in the median were more reliable than those of the mean, due to the influence of extreme values on the mean.

Bimodal data can cause problems for standard statistical tests and may occur commonly in phenological data. For instance, plankton blooms may occur in both spring and autumn in temperate regions (Edwards & Richardson, 2004), and many intertidal species have multiple spawning events (Moore *et al*., 2011). To deal with bimodality, Edwards & Richardson (2004) split the seasonal peaks into spring and autumn categories and analysed both as separate responses. Moore *et al*. (2011) used the 25th percentile to indicate the timing of spawning, thus avoiding biases in the mean spawning time caused by the bimodality of the data, but placing emphasis on first spawning peak.

While statistics based on the range centre statistics are popular for summarizing distribution data, it is important to consider which aspect of a range is most biologically relevant and provides the greatest ability to distinguish the effects of climate change. For instance, understanding the dynamics of the equatorward edge of a species’ range may be important for conservation of genetic diversity with global warming (Hampe & Petit, 2005). If the data were summarized using a centroid metric, this distinction may not be made. Multiple leading range edges, such as those for intertidal species on complex coastlines, may also provide greater opportunities for inferring the effects of climate change, because multiple observations of range shifts can be made for the same species within a reasonably small area.

A further consideration is that the study region usually does not cover the entire range of a species, particularly for cosmopolitan marine species (e.g. Perry *et al*., 2005; Hsieh *et al*., 2009; Nye *et al*., 2009). In this instance, the measured distribution centre does not provide a reliable estimate of the actual distribution centre. Most studies have addressed this issue by classifying species as being in the northern, southern or central parts of their ranges. Thus, the change in the mean observed distribution can be interpreted in terms of the biogeographical affinity of the species.

A final consideration for distribution shifts is whether to analyse purely the latitudinal component of a range shift, or the total distance of the range shift, which may be greater if the shift has a longitudinal component. In the oceans, temperature gradients are not strictly north–south, so species should not be expected to simply shift to higher latitudes in response to warming. For instance, the northern North Sea cools southwards, and species in this region may be moving towards the equator with ocean warming (Perry *et al*., 2005; Philippart *et al*., 2011). Thus, it may often be more meaningful to analyse the total distribution shift and report its direction in relation to prevailing temperature gradients and direction of warming in the region. Furthermore, some range shifts may be more evident as changes in the organism's depth distribution (Dulvy *et al*., 2008). While few datasets resolve depth (only four studies in the literature review analysed changes in depth), the potential for depth changes to hide horizontal distributional shifts should be considered, at least when formulating expectations.

### Community-wide studies

A major strength of Fodrie *et al*. (2010), as well as other examples above (Jenouvrier *et al*., 2005; Field *et al*., 2006; Hsieh *et al*., 2008; Genner *et al*., 2010), comes from the analysis of data from multiple species. In fact, 197 (69%) of the studies in our review reported data from more than one species. On ecological grounds, different species are expected to respond to climate change in different ways. Such differences could be expected between cold-water and warm-waters species or exploited and unexploited species. Analysis of community data thus gives researchers greater opportunities to test for changes that are consistent or inconsistent with climate change, relative to other sources of variability that may confound analyses based on single species.

Analyses of climate change impacts on communities can proceed with a combination of single-species analyses or with aggregated descriptors of community structure, such as diversity or multivariate statistics. In such studies, species-level impacts should also be reported, because they facilitate inclusion of results into syntheses. Without the reporting of species-level change, impacts of climate on some taxa may be underestimated by syntheses, or non-significant changes missed. For instance, the study of changes in distribution of 36 zooplankton species by Beaugrand *et al*. (2002) was included as only six assemblages in Parmesan & Yohe's (2003) meta-analysis because species-level changes were not reported in the original paper. This tendency towards reporting only assemblage-level changes may lead to a bias in reporting fewer but more consistent impacts for plankton communities compared with higher trophic levels, which are often analysed on a single-species basis. An additional consideration with community data is that phylogenetic similarity between species may result in similar responses to climate change. Controlling for phylogeny in studies of climate impacts is emerging as a powerful approach for understanding how species’ traits determine climate change responses (Davis *et al*., 2010).

Limitations on publication space in peer-reviewed journals may preclude inclusion of species-level impacts in the main body of a paper. Furthermore, competition to publish in the journals with the greatest impact also biases the published literature towards reporting positive results (Møller & Jennions, 2001); in the case of climate change ecology, this may mean over-representation of biological changes that are consistent with anthropogenic climate change. Both these biases are a serious problem for synthesis and for progressing the assessment of climate impacts on marine ecosystems. To overcome these issues, we recommend that the data or meta-data on species-level changes be provided in a repository, either as online supplementary material in the journal or an institutional repository (e.g. Table 2). This will assist interpretation of climate impacts and encourage re-analysis from different viewpoints.

**Table 2:** Information on some online data repositories

Data repository	Region	Type of data	Organization	Website
BlueNet	Australia	Marine science data	University of Tasmania	http://www.bluenet.org.au
Data Archive for Seabed Species and Habitats (DASSH)	United Kingdom	Benthic survey data	Marine Biological Association	http://www.dassh.ac.uk
DataOne	Global	All environmental data	DataOne	http://www.dataone.org
ICES* data centre	Global	Marine data, commercial catch records and marine meta-data	ICES	http://www.ices.dk/datacentre/Submissions/index.aspx
NCEAS† marine climate impacts working group	Global	Meta-data for marine biological impacts of climate change	NCEAS	https://groups.nceas.ucsb.edu/marine-climate-impacts/provide-data
NOAA‡ Data Center	Global	Oceanographic and marine biological data	NOAA	http://www.nodc.noaa.gov
Reef Base	Tropics	Coral reef ecosystem primary and meta-data	World Fish Centre	http://www.reefbase.org
Paleobiology database	Global	Occurrence and taxonomic data for any organism in any geological age	Multiple, collaborative	http://paleodb.org

* International Council for Exploration of the Sea.

† National Centre for Ecological Analysis and Synthesis.

‡ National Oceanic and Atmospheric Administration.

## Conclusions

We suggest that the issues discussed in this review should be considered when planning and conducting analyses in climate change ecology, and also when interpreting the reliability of published results from other studies. A summary of our suggestions is included below and are ordered roughly according to the sequence that they might be most useful. These suggestions are equally applicable to marine and terrestrial studies.

1. Consider how spatial and temporal resolution of data will influence the strength of inferences about drivers of change. For example, long time series with frequent observations, over large regions and over multiple climate cycles provide an ideal basis for interpreting recent anthropogenic climate change. Longer term palaeo-ecological data can also provide valuable baselines for assessing climate impacts.
2. Formulate alternative hypotheses for causal relationships between the ecological and climate variables. In some cases, observational studies can be coupled with experimental studies that shed light on the mechanisms driving change. In formulating alternative hypotheses, consider important drivers of ecological change, such as climate variability, ecosystem dynamics, other anthropogenic drivers of change (e.g. eutrophication, overfishing) and interactive effects. Where possible, data should be obtained on these drivers.
3. Identify response variables. Many different response variables may be derived from some datasets. The most statistically reliable response variables will generally have the largest sample size (e.g. using quantiles of distribution limits rather than the northerly most sighting of a single individual) and will be formulated to address the proposed hypotheses (e.g. north–south distributional changes may be irrelevant in regions with east–west currents). Non-conventional response variables may also reveal new patterns, such as considering changes in ecological variability rather than changes in the mean.
4. Formulate the identified processes as a statistical model or a series of models. Ideally, the models will include all drivers of change identified in step 2. Where possible, model-based approaches should be used rather than data transformations. Where temporal data cannot be obtained on key drivers, indirect approaches can be useful, such as comparisons among species. Furthermore, application of analytical methods beyond those traditionally used by ecologists (i.e. correlation and linear regression) will shed new light on the understanding of climate impacts. Promising methods rarely used in ecology include tests of cointegration, wavelets for the analysis of ecological cycles and spatio-temporal models.
5. Temporal autocorrelation should be considered in analysis if using time series data. Temporal autocorrelation patterns can often be reduced using filters, detrending or differencing. A more powerful approach for two variables can be to adjust the degrees of freedom in significance tests or to use a test of cointegration. If multiple predictors may influence the response, autoregressive models may be used and also allow estimation of rates of change.
6. Spatial autocorrelation and patterns should be considered if using spatial data. Spatial patterns can be ignored in analysis by grouping or averaging the data to a single value in space; however, this approach reduces the information content of the data. In some cases, meta-analysis, generalized additive models, mixed-effects models and geostatistics can be used to assist understanding the processes driving spatial patterns. Where spatial non-independence of data points cannot be accounted for by using covariates, it can be modelled explicitly. For spatially continuous data, models of spatial autocorrelation or spatial covariates can be used to account for non-independence of data points. Mixed-effects models can be used for data collected at discrete sites.
7. Metrics summarizing the rate-of-change for all species studied should be reported. Species-level metrics assist the uptake of the results of a study by other researchers and help in building global understanding of marine climate impacts. Registering data with an online database is encouraged (Table 2).

Consideration of these suggestions should help climate change ecologists apply appropriate statistical approaches to their data and afford them some confidence in the robustness of their results. We hope that this work will also encourage the re-analysis of archived datasets using appropriate approaches. A solid statistical basis for climate change ecology will help advance policy debates on climate change, improve predictions of impacts and aid the development of strategies for adaptive management.
